# N6-methyladenosine-modified TRIM37 augments sunitinib resistance by promoting the ubiquitin-degradation of SmARCC2 and activating the Wnt signaling pathway in renal cell carcinoma

**DOI:** 10.1038/s41420-024-02187-w

**Published:** 2024-09-30

**Authors:** Qiang Luo, Ting Dai, Yihong Dong, Jianpeng Liang, Zhipeng Xu, Zhixia Sun

**Affiliations:** 1 Department of Ultrasound, China-Japan Union Hospital of Jilin University, Changchun, 130000 Jilin China; 2 Harbin Medical University, Harbin, 150081 Heilongjiang China; 3 The First Peoples Hospital of Foshan, Foshan, 528000 Guangdong China; 4 Third Xiangya Hospital of Central South University, Changsha, 410013 Hunan China

**Keywords:** Renal cell carcinoma, Cancer therapeutic resistance, Methylation

## Abstract

Tripartite motif-containing 37 (TRIM37) is reportedly a key member of the superfamily of TRIM proteins. Emerging evidence underscores the close association between dysregulated TRIM37 expression and the progression of various human malignancies. However, the precise biological functions and regulatory mechanisms of TRIM37 remain elusive. This study aimed to elucidate the impact of TRIM37 on the chemotherapy sensitivity of renal cell carcinoma (RCC) and uncover its specific molecular regulatory role. Using RT-qPCR and western blot assays, we assessed TRIM37 expression in both RCC patients and RCC cells. Through in vitro and in vivo experiments, we investigated the effects of TRIM37 silencing and overexpression on RCC cell proliferation, stemness capacity, and chemotherapy sensitivity using colony formation and sphere formation assays. Additionally, a co-immunoprecipitation (Co-IP) experiment was conducted to explore putative interacting proteins. Our results revealed elevated TRIM37 expression in both RCC patient tumor tissues and RCC cells. Functional experiments consistently demonstrated that TRIM37 silencing reduced proliferation and stemness capacity while enhancing chemotherapy sensitivity in RCC cells. Furthermore, we discovered that TRIM37 mediates the degradation of SMARCC2 via ubiquitin-proteasome pathways, thereby further activating the Wnt signaling pathway. In conclusion, this study not only sheds light on the biological role of TRIM37 in RCC progression but also identifies a potential molecular target for therapeutic intervention in RCC patients.

## Introduction

Renal cell carcinoma (RCC) is the second most common malignancy of urological cancers with poor prognosis, and its incidence and mortality have experienced a significant surge in recent years [1, 2]. Based on the histopathological features, RCC can be categorized into three pathological subtypes, such as clear cell RCC (ccRCC), chromophobe RCC (chRCC), and papillary RCC (pRCC) [3]. Given the subtle onset of symptoms in RCC patients, many miss the opportunity for surgical intervention upon diagnosis [4, 5]. Moreover, due to the poor responsiveness of RCC to radiation and chemotherapy, effective treatment options are limited [6]. In light of these challenges, further exploration into RCC tumorigenesis holds promise for identifying potential therapeutic markers and enhancing clinical prognosis.

Trim37, a member of the Tripartite motif-containing (TRIM) superfamily [7], is characterized by three structural domains: RING finger, B-box, and coiled-coil [8]. A growing body of evidence suggests that TRIM37 is an oncogene upregulated in multiple malignancies, including pancreatic cancer [9], ovarian cancer [10], colorectal cancer, gastric cancer [11] and non-small cell lung cancer, involved in the regulation of promoting proliferation, epithelial-mesenchymal transition and apoptosis for tumor cells [12]. Previous reports revealed that TRIM37 harbored ubiquitin E3 ligase, which could regulate protein stability [13]. Notably, increased TRIM37 expression has been documented in hepatocellular carcinoma, where it orchestrates the degradation of P53 via the ubiquitin-proteasome pathway, influencing tumor progression [14]. Similarly, TRIM37, acting as an E3 ligase, has been implicated in K63-linked ubiquitination-mediated Activator protein-2 gamma (AP-2γ) degradation, facilitating breast cancer development [15]. However, few studies have hitherto focused on the biological function and role of TRIM37 in RCC, which is worthy of further study.

Herein, we found that TRIM37 expression was significantly upregulated in RCC tissue and correlated with poor prognosis. Comprehensive experiments demonstrated TRIM37’s role in governing the malignant phenotype and chemotherapy resistance in RCC in vitro and in vivo. Mechanistically, TRIM37 facilitates SMARCC2 degradation via the ubiquitin-proteasome pathway, thereby modulating the β-catenin/Wnt signaling axis and facilitating oncogenesis. These findings deepen our understanding of TRIM37’s molecular intricacies, shedding light on its potential function in RCC tumorigenesis and chemotherapy resistance. Consequently, TRIM37 has huge prospects as a diagnostic biomarker and viable therapeutic target for RCC patients.

## Result

### TRIM37 was associated with sunitinib resistance and poor prognosis in RCC

**Fig. 1:**
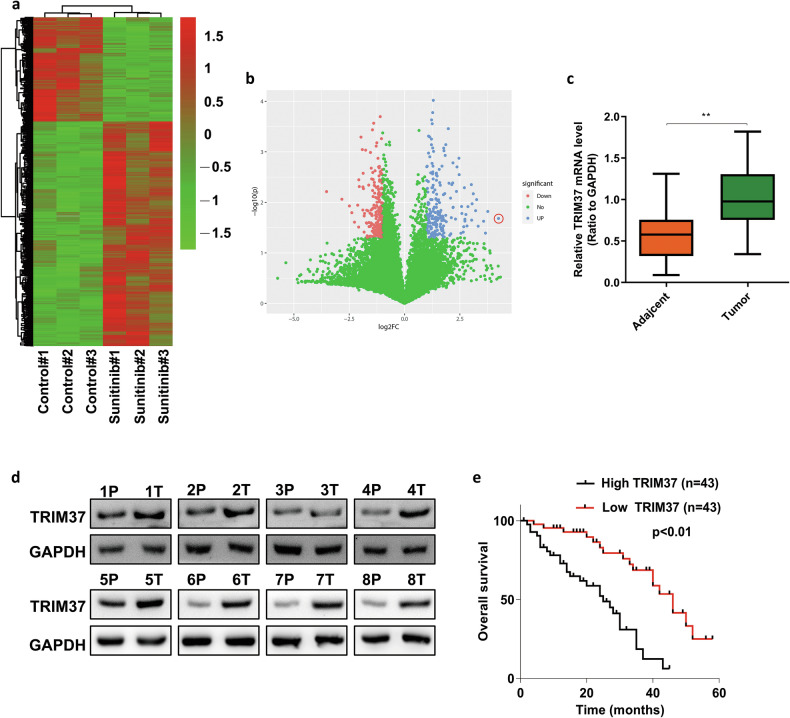
TRIM37 is highly expressed in RCC tumor tissue and predicts poor prognosis for RCC patients. **a** Heatmap showing the differentially expressed genes between Sunitinib treatment and Placebo treatment in the PDX mice model. **b** Volcano map showing differentially expressed genes. **c** RT-qPCR analysis of the mRNA of TRIM37 in tumor and adjacent tissue for patients with RCC. **d** The protein expression of TRIM37 was examined in RCC tumor and adjacent normal tissues by western blot. **e** Kaplan–Meier survival analysis was performed to show the association between the expression of TRIM37 and RCC patient prognosis. **p < 0.01.

Sunitinib, a multi-targeted tyrosine kinase inhibitor [16], is commonly used as a first-line therapy treatment for patients with advanced RCC. Despite its initial success, acquired therapeutic resistance often limits its efficacy. To uncover potential molecular factors associated with sunitinib resistance, we performed transcriptome sequencing on tumor tissue with sunitinib resistance and control tissue from a PDX mouse model (Fig. 1a), revealing significant upregulation of TRIM37 expression in sunitinib-resistant tissue (Fig. 1b). Further RT-qPCR analysis confirmed elevated TRIM37 mRNA expression in RCC tumor tissue compared to adjacent normal tissue (Fig. 1c). Moreover, Western blot analysis revealed that TRIM37 protein expression was higher in tumor samples (Fig. 1d). Notably, elevated TRIM37 expression was correlated with advanced TNM stage and distant metastasis (Table 1). Survival analysis demonstrated that higher TRIM37 expression correlated with poorer prognosis in RCC patients (Fig. 1e). Collectively, these findings indicate that TRIM37 may play a pivotal role in RCC tumorigenesis and progression.

**Table 1:** Relationship between TRIM37 and clinicopathological parameters in 86 RCC patients.

Variables	All cases	TRIM37 expression		*P*
		Low (*n* = 43)	High (*n* = 43)	
**Age (years)**				
<50	59	30	29	0.8162
≥50	27	13	14	
**Gendar**				
Male	54	28	26	0.6554
Female	32	15	17	
**AJCC stage T**				
T1/T2	67	38	29	0.0193
T3/T4	19	5	14	
**AJCC stage N**				
N0	81	41	40	ns
N1	5	2	3	
**AJCC stage M**				
M0	76	41	35	0.0436
M1	10	2	8	
**STAGE**				
I-II	61	35	26	0.0326
III-IV	25	8	17	
**GRADE**				
I-II	67	36	31	0.1937
III-IV	19	7	12	

### Overexpression of TRIM37 enhanced RCC cell proliferation and stemness

**Fig. 2:**
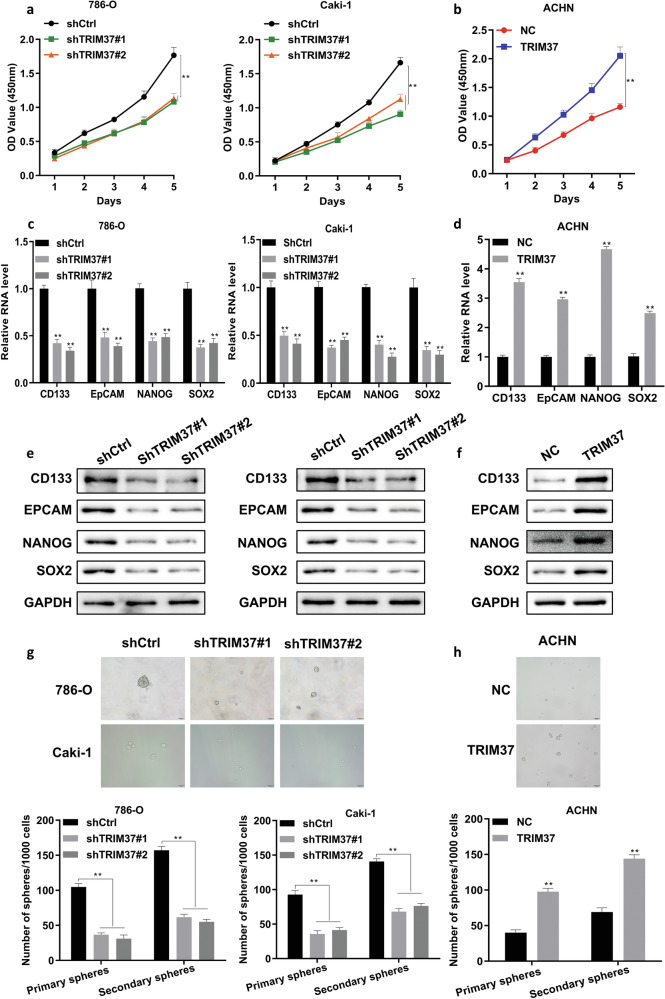
Overexpression of TRIM37 affected the biological behavior of RCC cells. **a**, **b** The effect of RCC cell proliferation was evaluated after TRIM37 silencing (**a**) or TRIM37 overexpression (**b**). **c, d** Effect of TRIM37 knockdown on 786-O and Caki-1 or TRIM37 overexpression ACHN cell stemness marker expression. **e, f** Analysis of the expression of stemness-related markers, including CD133, EpCAM, NANOG, and SOX2 in TRIM37 knockdown 786-O and Caki-1 cells, and TRIM37 overexpressed ACHN cell by Western blot assay. **g**, **h** The primary and secondary sphere formation capacities were detected in TRIM37 knockdown 786-O and Caki-1 cell, TRIM37 overexpressed ACHN cells (Top), and the representative images of secondary sphere formation in RCC cells (bottom). The data are represented as mean ± standard deviation (*n* = 3). **p < 0.01.

To elucidate TRIM37’s potential function in RCC tumorigenesis, we conducted both loss-of-function and gain-of-function assays. RT-qPCR and Western blot analyses showed significantly higher TRIM37 expression in RCC cell lines than in normal cell (Fig. S1a, b, c). The transfection efficiency of TRIM37 knockdown and overexpression was confirmed (Fig. S1d, e, f). Knockdown of TRIM37 notably inhibited the proliferation of Caki-1 and 786-O cells (Fig. 2a), while TRIM37 overexpression promoted ACHN cell growth (Fig. 2b) as determined by Cell Counting Kit-8 assays.

Additionally, TRIM37 knockdown resulted in reduced expression of stemness-related markers CD133, EPCAM, NANOG, and SOX2 (Fig. 2c), while TRIM37 overexpression increased their expression (Fig. 2d). This trend was consistently observed at the protein level (Fig. 2e, f). Suppression of TRIM37 led to decreased primary and secondary sphere formation in Caki-1 and 786-O cells (Fig. 2g), while TRIM37 overexpression enhanced sphere formation in ACHN cells (Fig. 2h). These findings collectively demonstrate that TRIM37 influences RCC cell proliferation and stemness.

### Silencing TRIM37 enhanced the Chemosensitivity of RCC Cells to Sunitinib in vitro and in vivo

**Fig. 3:**
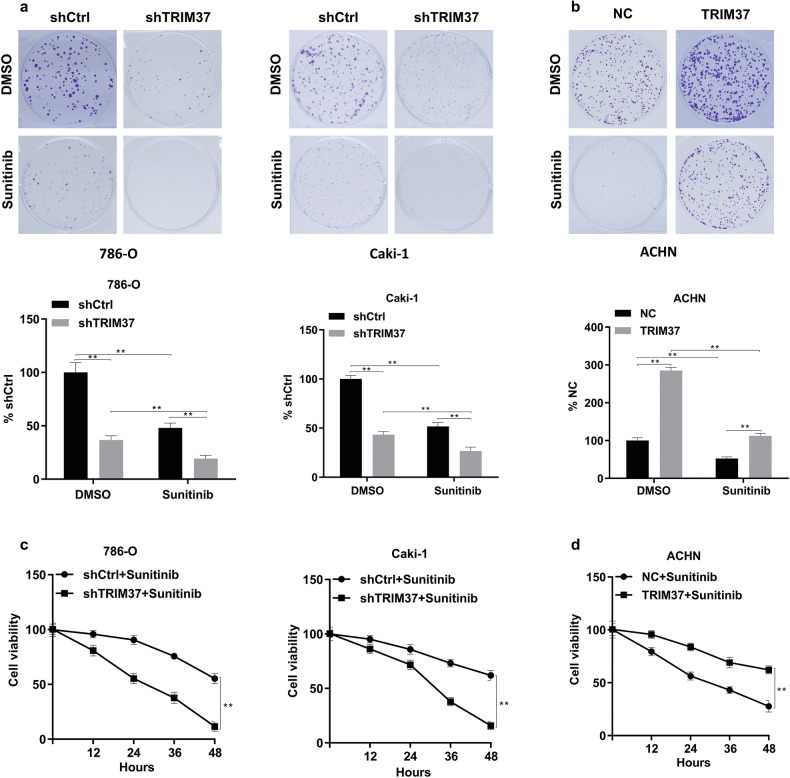
TRIM37 Overexpression suppressed the chemosensitivity of RCC cells to Sunitinib. **a** Colony formation in shTRIM37 silenced 786-O and Caki-1 cells after treatment with 30 μM Sunitinib. **b** Cell colony formation in TRIM37 overexpression plasmid-transfected ACHN cell after treatment with 30 μM Sunitinib **c** shTRIM37 silenced786-O and Caki-1 cell were treated with Sunitinib (30 μM) for 48 h. Cell viability was detected by MTT assay at different time points. **d** ACHN Cell viability was measured by MTT after treatment with Sunitinib (30 μM) for 48 h. The data are represented as mean ± standard deviation (*n* = 3). **p < 0.01.

**Fig. 4:**
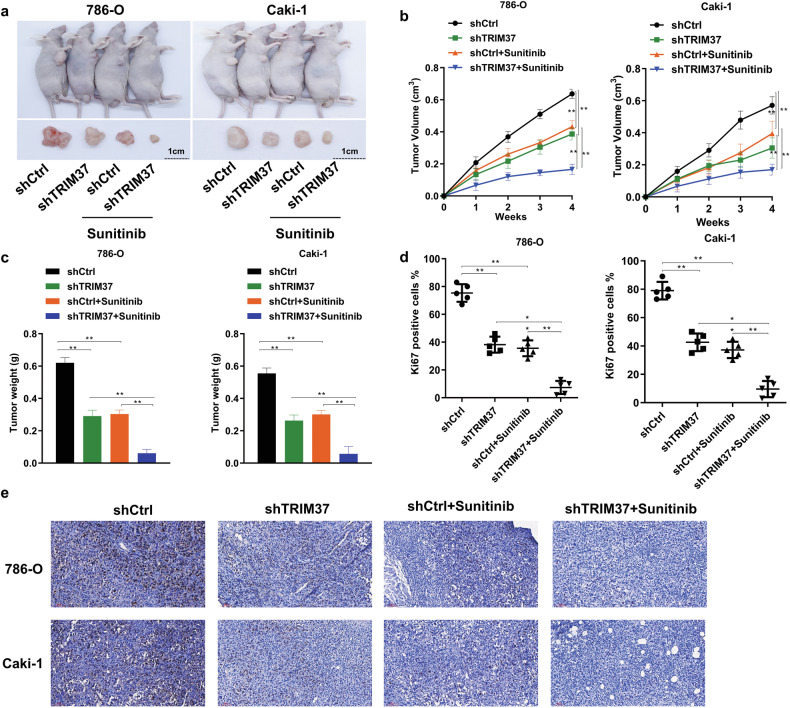
Silencing TRIM37 inhibited the growth of RCC cells and enhanced the Chemosensitivity of RCC Cells to Sunitinib. **a** Representative images of the subcutaneous xenograft tumors of 786-O and Caki-1 cell with stable shTRIM37 silencing in 4 weeks old nude mice following Sunitinib or saline treatment. **b**, **c** The xenograft tumor volume and weight in nude mice subcutaneously inoculated with 786-O or Caki-1 cell with stable shTRIM37 silencing or corresponding control cell for four weeks. **d** Graph showing ki67 expression. **e** The representative images of IHC for ki67 expression in different groups. The data are represented as mean ± standard deviation (*n* = 3). *p < 0.05, **p < 0.01.

Sunitinib resistance is a prevalent challenge in the treatment of RCC patients. To investigate the regulatory role of TRIM37 in sunitinib resistance, we conducted experiments involving the modulation of TRIM37 expression in RCC cells and the administration of appropriate concentrations of DMSO and sunitinib. As expected, TRIM37 knockdown significantly diminished sunitinib resistance in 786-O and Caki-1 cells (Fig. 3a), while an increase in resistance was observed in cells with TRIM37 overexpression (Fig. 3b). These findings suggest that TRIM37 is involved in the development of chemotherapy resistance to sunitinib in RCC cells. Furthermore, to validate these results, we assessed cell viability at different time points following exposure to 30 μM sunitinib. The outcomes demonstrated that silencing TRIM37 enhanced the sensitivity of RCC cells to sunitinib (Fig. 3c), while TRIM37 overexpression observed the opposite result (Fig. 3d). An in vivo experiment was conducted to explore the potential impact of TRIM37 on the growth capacity and sunitinib resistance of RCC cells by establishing a subcutaneous xenotransplant tumor model in nude mice. The results revealed a substantial deceleration in the rate of RCC cell growth upon TRIM37 silencing, accompanied by a more pronounced anti-tumor effect of sunitinib compared to control cells (Fig. 4a). Consistent data regarding tumor volume and weight further supported the notion that decreased TRIM37 expression curbs RCC cell growth (Fig. 4b, c). Additionally, we observed that the combination of TRIM37 silencing and sunitinib treatment yielded the lowest expression of Ki67, a common cell proliferation marker, compared to other single interventions (Fig. 4d, e). Collectively, these results indicate that silencing TRIM37 not only suppresses RCC growth but also enhances the therapeutic efficacy of sunitinib.

### TRIM37 activates the Wnt signaling pathway

**Fig. 5:**
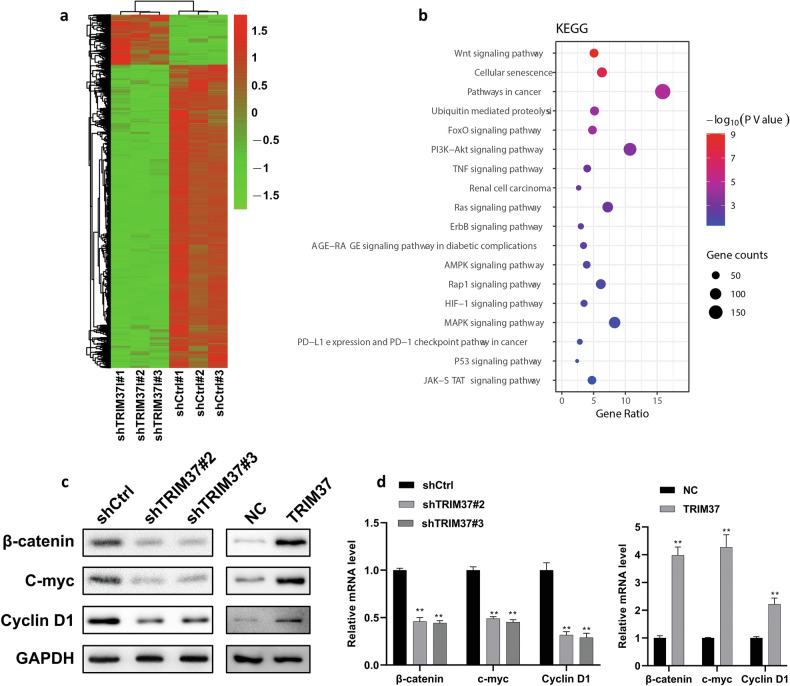
TRIM37 affected the progression of RCC via activating the Wnt signaling pathway. **a** Heatmap showing differential gene expression after TRIM37 silencing in 786-O cells. **b** The KEGG analysis was performed to recognize the significantly enriched pathways after TRIM37 silencing. **c** The expression of Wnt pathway-related proteins, including β-catenin, C-myc, and Cyclin D1, was measured by western blot after TRIM37 silencing in 786-O or TRIM37 overexpression in ACHN cells. **d** Bar chart representing the mRNA of β-catenin, C-myc, and Cyclin D1. The data are represented as mean ± standard deviation (*n* = 3). **p < 0.01.

To gain deeper insights into the molecular mechanisms underlying TRIM37’s influence on the malignant behavior of RCC, we conducted RNA transcriptome sequencing on RCC cells transfected with shTRIM37 or scrambled control (Fig. 5a). Analysis of the resulting data revealed a total of 6217 differentially expressed genes, including 5253 downregulated and 964 upregulated unique mRNAs. Subsequent Kyoto Encyclopedia of Genes and Genomes (KEGG) analysis provided further insights by demonstrating significant enrichment of these differentially expressed genes in multiple cancer-related signaling pathways, with emphasis on the Wnt signaling pathway (Fig. 5b). As previously reported, the Wnt signaling pathway is a conserved pathway with critical roles in growth, development, and metabolism. Dysregulation of this pathway is frequently implicated in tumor development and linked to conditions like obesity and diabetes [17]. To corroborate TRIM37’s potential involvement in RCC development, we evaluated the mRNA and protein expression levels of Wnt pathway-related proteins using RT-qPCR and Western blot analyses. These results exhibited downregulation of β-catenin, C-myc, and Cyclin D1 following TRIM37 silencing, whereas TRIM37 overexpression induced the expression of proteins associated with the pathway (Fig. 5c, d). Our findings suggest that TRIM37 exerts its regulatory influence on RCC development by activating the Wnt signaling pathway.

### TRIM37 downregulated SMARCC2 expression by mediating ubiquitin-proteasome degradation

**Fig. 6:**
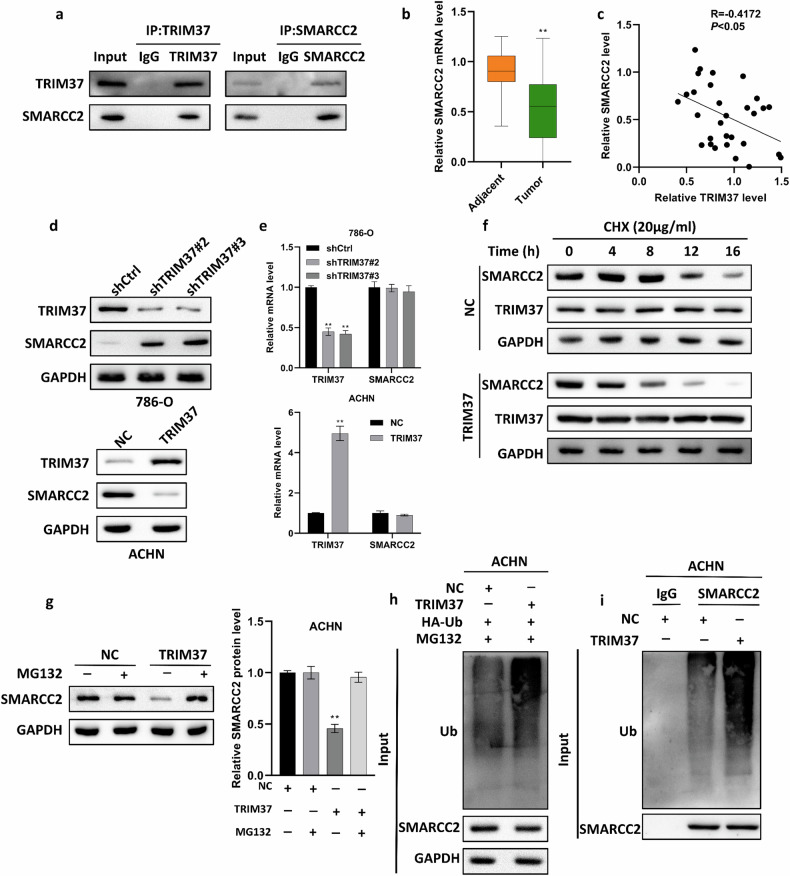
TRIM37 decreased the stability of SMARCC2 by increasing ubiquitin degradation. **a** Co-IP experiment demonstrating the interaction of TRIM37 and SMARCC2. **b** The mRNA of SMARCC2 was measured in tumor and adjacent tissue for 30 RCC patients. (**c**) Analysis of the correction of TRIM37 level and SMARCC2 level. **d**, **e** The protein and mRNA levels of SMARCC2 were separately measured after Trim37 silencing and TRIM37 overexpression. **f** ACHN cells were transfected with TRIM37 plasmids for 24 h and then incubated with 20 μM cycloheximide at different times. **g** ACHN cells transfected TRIM37 and NC plasma were treated with 5 μM MG132 for 4 h, and western blot detected the SMARCC2 protein level. **h** TRIM37 overexpression in ACHN cells simultaneously co-transfected with HA-tagged ubiquitin-expressed plasmid. After treatment with 5 μM MG132 for 4 hours, an Anti-HA antibody was used to conduct the immunoprecipitation assay and cell proteins were used for western blotting. **i** The cell lysates were used to Co-IP with the SMARCC2 antibody, followed by western blotting. The data are represented as mean ± standard deviation (*n* = 3). **p < 0.01.

To determine the potential molecular mechanisms regulated by TRIM37, we conducted a comprehensive analysis of proteins that closely interact with TRIM37 by integrated Co-IP experiments with mass spectrometry analysis. First, we identified SMARCC2 as a molecule that could interact with TRIM37 and further confirmed the association by western blot (Fig. 6a). Next, we quantified the expression of SMARCC2 in RCC and found SMARCC2 exhibited lower expression in tumors compared with normal tissue (Fig. 6b). Correlation analysis showed that TRIM37 expression was negatively correlated with SMARCC2 (Fig. 6c). Subsequently, we observed the potential effect of TRIM37 on the mRNA and protein level of SMARCC2. Silencing TRIM37 could significantly decrease the protein levels of SMARCC2 but not the SMARCC2 mRNA expression. Conversely, TRIM37 overexpression upregulated SMARCC2 protein expression but did not affect the mRNA level (Fig. 6d, e), suggesting that TRIM37 decreased the protein level of SMARCC2 at the post-transcriptional level. Since ubiquitination modification is one of the most common protein modifications, and TRIM37 has been identified as an E3 ubiquitination enzyme in previous studies, we hypothesized that TRIM37 could cause the downregulation of SMARCC2 expression by promoting the ubiquitination degradation of the SMARCC2 protein. To validate this hypothesis, we observed the impact of TRIM37 overexpression on the half-life of SMARCC2 in the presence of de novo protein synthesis inhibitor cycloheximide. We found that TRIM37 overexpression shortened the half-life of SMARCC2 (Fig. 6f). Furthermore, to explore whether TRIM37 regulates SMARCC2 stability via ubiquitination degradation, we treated ACHN cells with potent cell-permeable reversible proteasome inhibitor MG132. Importantly, MG132 administration rescued the destabilizing effect of TRIM37 on SMARCC2 (Fig. 6g). In addition, we detected the ubiquitination expression level and found that overexpression of TRIM37 could increase the expression of SMARCC2 in the presence of MG132 (Fig. 6h, i). Taken together, the above findings suggest that TRIM37 can destabilize the SMARCC2 protein mediated by ubiquitination-proteasome degradation.

### Trim37 regulation of SMARCC2 effects and promotion of tumorigenesis via the Wnt signaling pathway

**Fig. 7:**
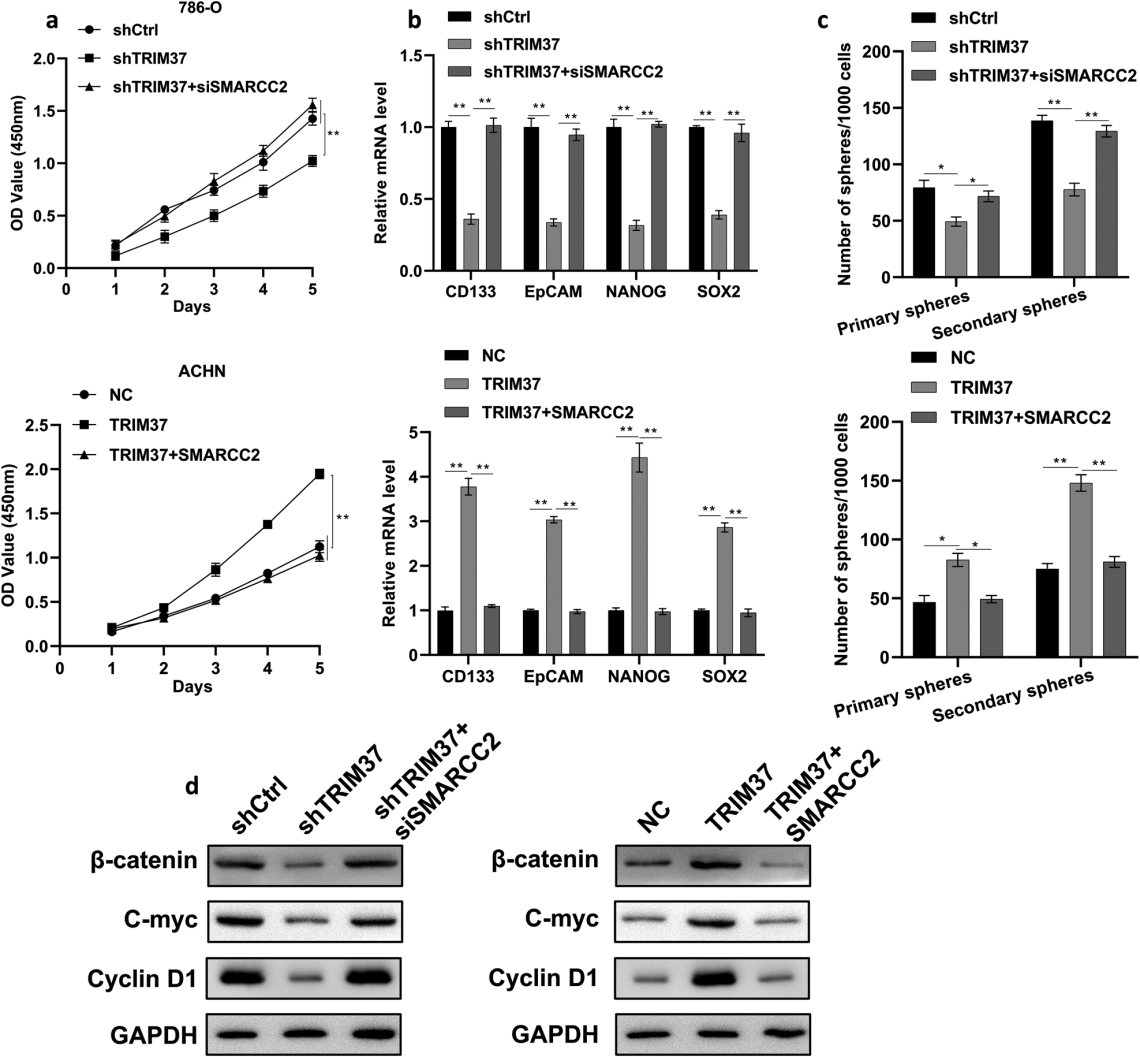
Trim37 regulated the effects of SMARCC2 to promote tumorigenesis via the Wnt signaling pathway. **a** CCK-8 assays showing the RCC cell proliferation ability after co-transfection of TRIM37 silencing and knockdown of SMARCC2 (top) and TRIM37 and SMARCC2 overexpression (bottom). **b** RT-qPCR assay showing the mRNA expression of stemness marker in 786-O and ACHN cell. **c** The primary and secondary sphere formation capacities were separately analyzed with different co-transfection experiments with TRIM37 and SMARCC2. **d** Western blot showing the protein level of the Wnt signaling pathway. The data are represented as mean ± standard deviation (*n* = 3). *p < 0.05, **p < 0.01.

Building upon the above findings, we conducted a series of rescue experiments to elucidate the potential role of SMARCC2 in TRIM37-mediated RCC cell malignancy. CCK8 assay results revealed that the inhibitory effect of shTRIM37 on RCC cell proliferation was significantly counteracted by SMARCC2 silencing, while SMARCC2 overexpression attenuated the growth induced by TRIM37 overexpression (Fig. 7a). Moreover, the reduction in stemness molecular markers mediated by shTRIM37 was restored by inhibiting SMARCC2 expression, leading to a substantial increase in related stemness marker levels (Fig. 7b). Additionally, SMARCC2 silencing significantly inhibited the primary and secondary sphere formation capacities induced by shTRIM37 transfection in 786-O cells. Conversely, the enhanced sphere formation capability resulting from TRIM37 overexpression was effectively reversed upon SMARCC2 overexpression (Fig. 7c). Notably, a previous study reported that SMARCC2 can suppress Wnt/β-catenin signaling, inhibiting glioma cell migration and invasion. However, its role in regulating the Wnt signaling pathway in RCC remained unclear. We observed that TRIM37 silencing reduced the expression of β-catenin, C-myc, and Cyclin D1, which could be restored to some extent by SMARCC2 knockdown. Moreover, the upregulation of SMARCC2 could rescue the effects of TRIM37 overexpression on Wnt signaling pathway-related proteins (Fig. 7d). In summary, these findings suggest that TRIM37 affects RCC tumorigenesis by modulating the expression of SMARCC2 and activating the Wnt signaling pathway.

### m6A modification mediated by METTL3 upregulated the expression of TRIM37

**Fig. 8:**
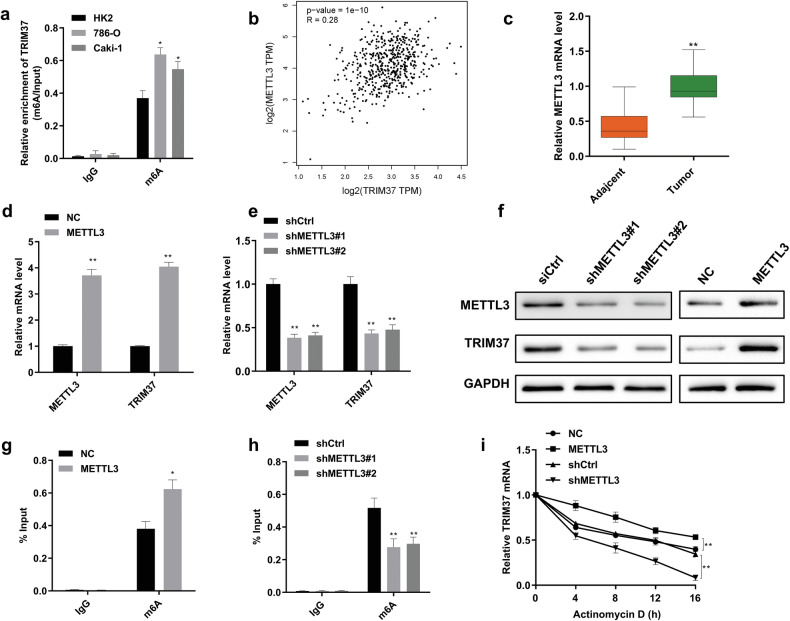
m6A modification mediated METTL3 affected the expression of TRIM37. **a** The enrichment of m6A modification-mediated TRIM37 was measured by the RIP and RT-qPCR assay in RCC and normal cells. **b** The positive correlation between the expression level of TRIM37 and METTL3 was confirmed in TCGA database. **c** METTL3 expression in HCC was analyzed in 30 pairs of RCC patients. **d**, **e** The effect of METTL3 overexpression or knockdown on TRIM37 mRNA expression. **f** Analysis of the effect of METTL3 overexpression or knockdown on TRIM37 protein expression. **g, h** RIP-RT-qPCR assay showing the enrichment expression of m6A modified TRIM37 following METTL3 overexpression or METTL3 depletion. **i** TRIM37 stability analysis in 786-O cells with METTL3 overexpression and METTL3 depletion in the presence of actinomycin D. The data are represented as mean ± standard deviation (*n* = 3). *p < 0.05, **p < 0.01.

Current evidence suggests that the dysregulation of N6-methyladenosine (m6A) is closely linked to tumorigenesis [18]. m6A-modified RNA is specifically recognized and bound by m6A recognition proteins, exerting influence on RNA expression and regulating various biological processes in physiological and pathological contexts [19]. Numerous m6A sites were predicted to interact with TRIM37 through the RMBase database (http://rna.sysu.edu.cn/rmbase/index.php). Furthermore, m6A levels were higher in 786-O and Caki-1 cells compared to normal HK2 cell, as determined by RIP and RT-qPCR assays (Fig. 8a). Intriguingly, METTL3 expression positively correlated with TRIM37 levels in RCC tissue (Fig. 8b). METTL3, a common methyltransferase, is upregulated in various tumors to regulate tumor progression [20]. Additionally, our investigation revealed upregulation of TRIM37 in RCC tumor samples compared to corresponding adjacent tissues (Fig. 8c). Consistent with these findings, Western blot and RT-qPCR analyses demonstrated that silencing METTL3 decreased TRIM37 expression, whereas METTL3 overexpression remarkably enhanced TRIM37 expression (Fig. 8d–f). Moreover, RIP assays indicated that METTL3 deletion reduced m6A modification of TRIM37 in RCC cells, while METTL3 overexpression yielded the opposite effect (Fig. 8g, h). Finally, in the presence of actinomycin D, a known inhibitor of de novo RNA synthesis, METTL3 overexpression reduced TRIM37 stability, while METTL3 silencing had the opposite effect (Fig. 8i). Collectively, these findings underscore the pivotal role of METTL3 in upregulating TRIM37 expression during RCC development.

## Discussion

Current evidence suggests that RCC is the most prevalent renal malignancy, marked by its high lethality and unfavorable prognosis [21]. Despite continuous strides in treatment methods, the efficacy of conventional therapies for RCC, especially in advanced cases, remains less promising [22]. Currently, targeted therapy is a optimal treatment choice for RCC patients [23]. In particular, Sunitinib is recognized as a first-line therapy treatment for patients with advanced RCC [24, 25]. Nevertheless, a considerable number of patients develop resistance to Sunitinib, resulting in disease progression and ultimately affecting patient survival [6]. Therefore, there is an urgent need to focus on gaining a thorough comprehension of the onset and progression of RCC, a profound insight into its underlying causes, a underlying mechanism of sunitinib resistance and the exploration of novel treatment objectives for its prevention.

The TRIM protein family has been closely linked to the emergence and progression of diverse malignant tumors [26]. Among them, TRIM37 is a significant member. While prior investigations have highlighted TRIM37’s involvement in modulating various biological behaviors of human malignancies [27], until now, only one study has reported that it mediates migration, invasion and EMT process in RCC cells [28]. However, its role in chemotherapy resistance of RCC has remained relatively unexplored. In this study, we meticulously manipulated TRIM37 expression in RCC cells to systematically scrutinize its impact on proliferation, stemness, and chemotherapy resistance. Our findings revealed significant upregulation of TRIM37 expression in sunitinib-resistant tissue, and elevated TRIM37 expression in both RCC tumors and cells, the high level of TRIM37 was associated with advanced TNM stage, distant metastasis and poor prognosis. These observation is in accordance with previous study indicating that it plays a cancer-promoting role [28]. Furthermore, our data underscored for the first time that inhibiting TRIM37 could curtail RCC cell proliferation, dampen sphere-forming capacity, and heighten Sunitinib sensitivity. These results, consistent with the literature [28], substantiated TRIM37’s pro-cancer influence on RCC progression and its role in chemotherapy resistance, reinforcing our conclusions’ robustness.

It has been established that SWItch/sucrose non‑fermentable (SWI/SNF) is a tumor suppressor that regulates epithelial‑mesenchymal transition [29, 30]. Moreover, SMARCC2 is the core subunit of the SWI/SNF complex [31], which is prone to mutation in various malignant cancers, resulting in low expression levels [32, 33]. Our investigation unveiled SMARCC2 as a potential interacting partner of TRIM37 through CO-IP assays and mass spectrometry. Furthermore, SMARCC2 exhibited diminished expression in tumor tissue, with a negative correlation to TRIM37 levels. We next demonstrated that TRIM37 promoted SMARCC2 degradation by potentiating the ubiquitination pathway. Collectively, our data illuminated TRIM37’s role in modulating SMARCC2 expression in the context of RCC.

Previous research has extensively expounded on Wnt-mediated signaling pathways and their influence on embryonic development, tissue homeostasis, and tumorigenesis [34]. In this respect, SMARCC2 has been implicated in mediating the Wnt/β-catenin signaling pathway to suppress glioma cell migration and invasion through interaction with C-MYC [35]. Our KEGG pathway analysis consistently highlighted the significant role of the Wnt signaling pathway in TRIM37-mediated signaling transduction. Remarkably, our data further underscored TRIM37’s regulatory role in Wnt signaling, primarily through its control over SMARCC2 expression. Collectively, our findings elucidate how SMARCC2 acts as an intermediary in transmitting TRIM37’s effects within RCC via modulation of the Wnt signaling pathway.

Recent studies have shown that m6A modification plays a crucial role in the occurrence and progression of tumors [36]. We further explored that TRIM37 overexpression in RCC is mainly due to its stability mediated by m6A modification. METTL3, as an important N6-methyladenosine (m(6)A) regulator, exhibites abnormal expression in multiple malignances and promotes tumor progression [37, 38]. Here, our results show that METTL3 mediates TRIM37 m6A modification to enhance TRIM37 stability, and further promote sunitinib resistance.

**Fig. 9:**
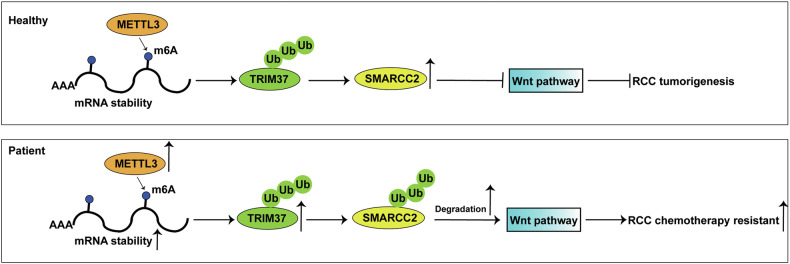
A schematic diagram of the role of TRIM37 in RCC and its mechanism. METTL3-mediated m6A methylation stabilizes and upregulates TRIM37 expression. TRIM37 as a oncogenic molecule induces SMARCC2 degradation through ubiquitination, thereby further activating the Wnt signaling pathway, thus contributing to the RCC tumorigenesis and chemotherapy resistance.

Overall, our study findings provide compelling evidence of TRIM37’s pivotal role in driving RCC cell malignancy and chemotherapy resistance through orchestrating SMARCC2 expression and modulating the Wnt signaling pathway (Fig. 9). These insights cast TRIM37 as a potential novel target to improve the treatment and prognosis prediction of RCC patients.

## Method

### Patients and Cell lines

86 pairs of tumor and corresponding adjacent tissue specimens were obtained from RCC patients who underwent surgical therapy at China-Japan Union Hospital of Jilin University. Fresh tissue samples were rapidly stored in liquid nitrogen after surgical excision. Informed consent was obtained from all patients, and the study’s design was reviewed and approved by the Ethics Committee of China-Japan Union Hospital of Jilin University.

The RCC cell lines used in the study were obtained from the ATCC (American Type Culture Collection). Specifically, 786-O cells were cultured in an RPMI-1640 medium, while ACHN, A498, and HK2 cells were cultured in a DMEM medium. The respective media were supplemented with 10% fetal bovine serum and 1% penicillin-streptomycin. All cells were cultured in a 37 °C incubator with 5% CO_2_.

### Western blot

Total protein was extracted from tissues and cells using a potent cell lysate, and protein concentration was quantified using the BCA method. Equal amounts of protein were subjected to SDS-PAGE, followed by transfer to polyvinylidene difluoride (PVDF) membranes. After blocking in 5% skim milk for one hour, PVDF membranes were incubated with primary and secondary antibodies. Protein expression was analyzed using electrochemiluminescence imaging (Bio-Rad, USA).

### RT-qPCR assay

Total RNA was extracted from tissues and transfected cells using TRIzol reagent (Invitrogen) according to the manufacturer’s protocol. Reverse transcription was performed on 1 μg of total RNA using a reverse transcription kit (Takara) to generate cDNA. SYBR Premix Ex Taq II (Takara) was used for qPCR analysis. Primer sequences are provided in supplementary table 2.

### Cell transfection

TRIM37 short hairpin RNA (shTRIM37), SMARCC2 small interfering RNA (siSMARCC2), pcDNA3.1 vectors expressing TRIM37 or SMARCC2, and their corresponding scramble controls were obtained from GeneChem (China). Cells were transfected with the respective siRNA or plasmids using Lipofectamine 3000 (Thermo Fisher Scientific) when cell density reached 70-80% in a six-well plate. Stable cell lines expressing TRIM37 and scramble control were generated by culturing in 0.5 μg/ml puromycin for ten days following infection. Sequences are provided in supplementary table 3.

### Cell counting Kit-8 (CCK8)

1×10^3^ RCC cells that underwent transfection with shRNA or plasma were incubated in a 96-well plate and then cultured in a 37 °C incubator. In brief, 10 μL of CCK-8 reaction reagent (Dojindo) was supplemented to each well and incubated for one hour at 37 °C. Finally, the absorbance value (OD) was further analyzed at a wavelength of 450 nm.

### Colony formation assay

RCC cells (1×10^3^ cells/well) were seeded in a six-well plate and cultured at 37 °C with 5% CO_2_ for 1-2 weeks. After fixing with 4% cell fixative, cells were stained with crystal violet (Solarbio) for one hour. Colonies with more than fifty cells were counted and analyzed.

### Sphere formation assay

RCC cells were cultured at a density of 1000 cells/well in ultralow attachment six-well plates (Corning) for one week in DMEM/F12 medium (Invitrogen) supplemented with B27 (1:50, GIBCO), 20 ng/ml EGF (Sigma), and 20 ng/ml bFGF (Sigma). Primary spheres were collected, dissociated, and reseeded to produce secondary spheres for one more week. The number of spheres was counted under a microscope.

### Co-immunoprecipitation (Co-IP) assay

Fresh cell protein was collected for immunoprecipitation. The target antibody was incubated with protein A/G magnetic beads (MCE, New Jersey) at 4 °C overnight. The antibody-coated beads were then incubated with protein, followed by IP buffer washes. Eluted proteins were used for subsequent experiments.

### Protein half-life assay

RCC cells were treated with cycloheximide (20 μM) to inhibit protein synthesis and collected at different time points for Western blot analysis.

The establishment of a subcutaneous xenograft model and patient-derived tumor xenograft model (PDX) in mice.

Related animal experiments were reviewed and approved by the Animal Experiment Ethics Committee of the First Hospital of Jilin University. Stably downregulated TRIM37 786-O and Caki-1 cells and their scramble controls were subcutaneously injected into 4–6 weeks old male nude mice (*n* = 6 mice per group). Tumor volume was monitored, and when tumors reached 200 mm^3^, mice were treated with sunitinib (40 mg/kg) twice a week. Tumors were collected after four weeks.

Fresh tumor fragments were subcutaneously transplanted into 4–6 weeks old male NOD scid gamma mice. After completing a sunitinib treatment cycle, tumor tissue was transplanted into new NOD mice, followed by sunitinib treatment. Third-generation xenografts were finally obtained.

### Statistical analysis

Data from three independent experiments were presented as mean ± standard deviation (SD). GraphPad Prism 9.5.1 was used for statistical analysis. Student’s t-test and one-way analysis of variance were used for group comparisons. Pearson’s correlation analysis was conducted to identify gene expression correlations. *p* value < 0.05 was statistically significant.

## Supplementary information

Supplementary Figure 1

Supplementary table

Supplemantary material of WB

## Data Availability

The article includes all the data supporting the conclusions of this manuscript. The corresponding author can be contacted for raw data requests.
