# Supplementary material for: N6-methyladenosine-modified TRIM37 augments sunitinib resistance by promoting the ubiquitin-degradation of SmARCC2 and activating the Wnt signaling pathway in renal cell carcinoma
Source: Cell Death Discov. 2024 Sep 30;10:418. doi: 10.1038/s41420-024-02187-w (PMC11442835; doi:10.1038/s41420-024-02187-w)
Supplement: Supplementary file 1 — Supplementary Figure 1 [file 41420_2024_2187_MOESM1_ESM.docx]

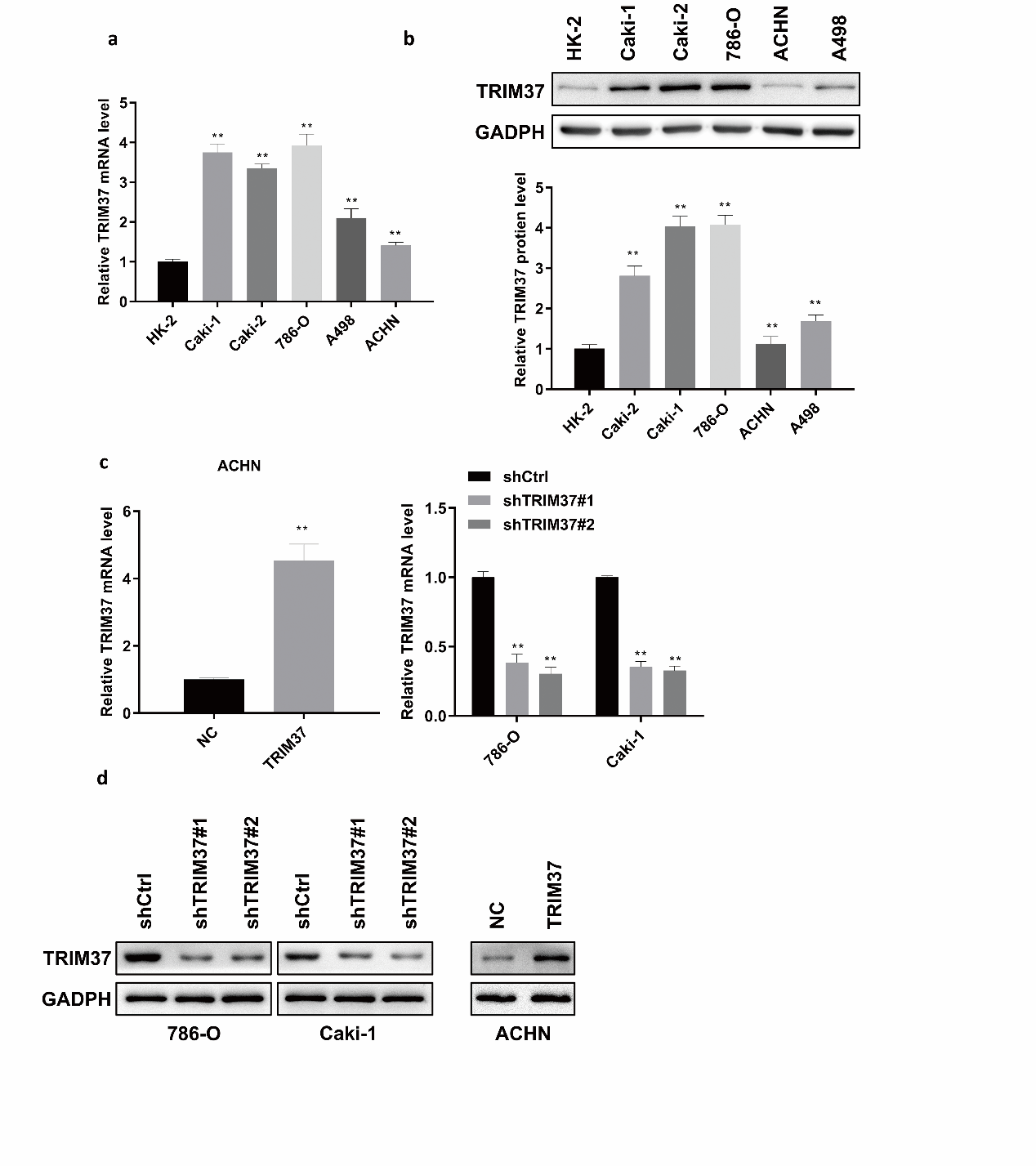


Supplementary Fig. 1

The expression and transfection efficiency of TRIM37 in RCC cells. (a) TRIM37 mRNA levels were detected in five RCC cells and normal renal cells. (b) Analysis of TRIM37 expression in five RCC cells and normal renal cells. (c, d) Transfection efficiency of TRIM37 in protein and mRNA level after overexpression and silencing of TRIM37. **p < 0.01.
