# Supplementary material for: N6-methyladenosine-modified TRIM37 augments sunitinib resistance by promoting the ubiquitin-degradation of SmARCC2 and activating the Wnt signaling pathway in renal cell carcinoma
Source: Cell Death Discov. 2024 Sep 30;10:418. doi: 10.1038/s41420-024-02187-w (PMC11442835; doi:10.1038/s41420-024-02187-w)
Supplement: Supplementary file 2 — Supplementary table [file 41420_2024_2187_MOESM2_ESM.docx]

**Supplementary table**

**Supplementary table 1: Cell lines**

| Name | Source | Cat# |
| --- | --- | --- |
| HK2 | ATCC | CRL-2190 ™ |
| Caki-1 | ATCC | HTB-46 ™ |
| Caki-2 | ATCC | HTB-47 ™ |
| 786-O | ATCC | CRL-1932 ™ |
| ACHN | ATCC | CRL-1611 ™ |
| A498 | ATCC | HTB-44 ™ |

**Supplementary table 2: The information of primers used in the study**

| Gene name |  | Sequece (5’—3’) |
| --- | --- | --- |
| GAPDH | Forward | GGAGCGAGATCCCTCCAAAAT |
|  | Reverse | GGCTGTTGTCATACTTCTCATGG |
| TRIM37 | Forward | TATGGAGAAATTGCGGGATGC |
|  | Reverse | GTCAGCCAGCGCCTAATACAG |
| SMARCC2 | Forward | AGTGCCAACCCCTTCAC |
|  | Reverse | GCTCAGGCATCAGGAGAC-3 |
| METTL3 | Forward | AGATGGGGTAGAAAGCCTCCT |
|  | Reverse | TGGTCAGCATAGGTTACAAGAGT |

**Supplementary table 3: The information of short interfering RNA or small interfering RNA used in the study**

| Short interfering RNA | | Sequence information | Complany |
| --- | --- | --- | --- |
| shTRIM37 | shTRIM37#1 | 5'‑GCTGAAGAATAAGCTTATA‑3' | Gene Parma  (Shanghai, China) |
|  | shTRIM37#2 | 5'‑GCTACGAGAACTAGTAAAT‑3' |  |
| siSMARCC2 | siCtrl | 5'‑TTUUGAACCAAGAAGCCUCCC‑3' | Gene Parma  (Shanghai, China) |
|  | siSMARCC2 #1 | 5'‑CUCGGCAAGAACUACAAGATT‑3'  5'‑UCUUGUAGUUCUUGCCGAGTT‑3' |  |
|  | siSMARCC2 #2 | 5'‑CUCGGCAAGAACUACAAGATT‑3'  5'‑UCUUGUAGUUCUUGCGAGTT‑3' |  |
| siMETTL3 | siMETTL3#1 | 5'‑GCUGCACUUCAGACGAAUUTT‑3'  5'‑AAUUCGUCUGAAGUGCAGCTT‑3' | Gene Parma  (Shanghai, China) |
|  | siMETTL3#2 | 5'‑GCUCAACAUACCCGUACUATT‑3'  5'‑UAGUACGGGUAUGUUGAGCCT‑3' |  |
|  | siMETTL3#3 | 5'‑GCAAGAAUUCUGUGACUAUTT‑3'  5'‑AUAGUCACAGAAUUCUUGCAC‑3' |  |

**Supplementary table 4: Primary antibodies for western blot**

| Antibodies | Source | Cat# |
| --- | --- | --- |
| Anti-TRIM37 antibody | Abcam | ab264190 |
| Anti-GAPDH antibody | Abcam | ab8245 |
| Anti-CD133 antibody | Cell signaling technology | 64326 |
| Anti-EpCAM antibody | Cell signaling technology | 2929 |
| Anti-NANOG antibody | Cell signaling technology | 4903 |
| Anti-SOX2 antibody | Cell signaling technology | 23064 |
| Anti-β-catenin antibody | Cell signaling technology | 8480 |
| Anti-C-myc antibody | Cell signaling technology | 5605 |
| Anti-Cyclin D1 antibody | Cell signaling technology | 55506 |
| Anti-SMARCC2 antibody | Cell signaling technology | 12760 |
| Anti-METTL3 antibody | Cell signaling technology | 86132 |
