# Supplementary material for: N6-methyladenosine-modified TRIM37 augments sunitinib resistance by promoting the ubiquitin-degradation of SmARCC2 and activating the Wnt signaling pathway in renal cell carcinoma
Source: Cell Death Discov. 2024 Sep 30;10:418. doi: 10.1038/s41420-024-02187-w (PMC11442835; doi:10.1038/s41420-024-02187-w)

Unprocessed Wb image for Figure 1

Fig.1d

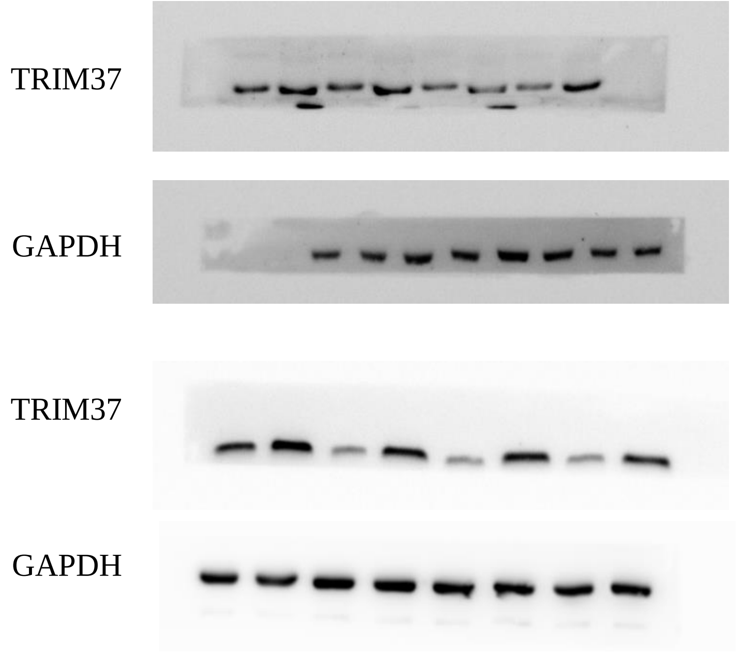

Unprocessed Wb image for Figure S1

Fig.S1b

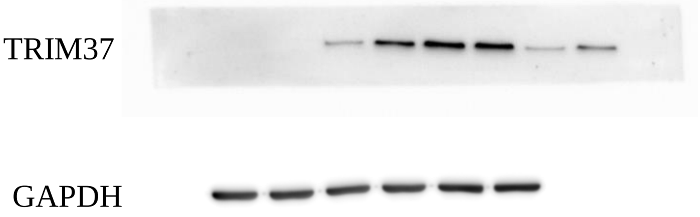

Fig.S1d

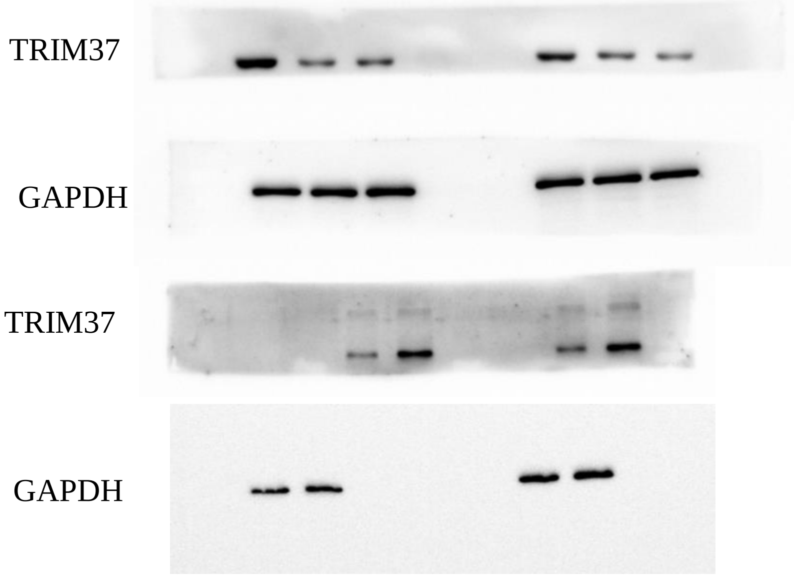

Unprocessed Wb image for Figure 2

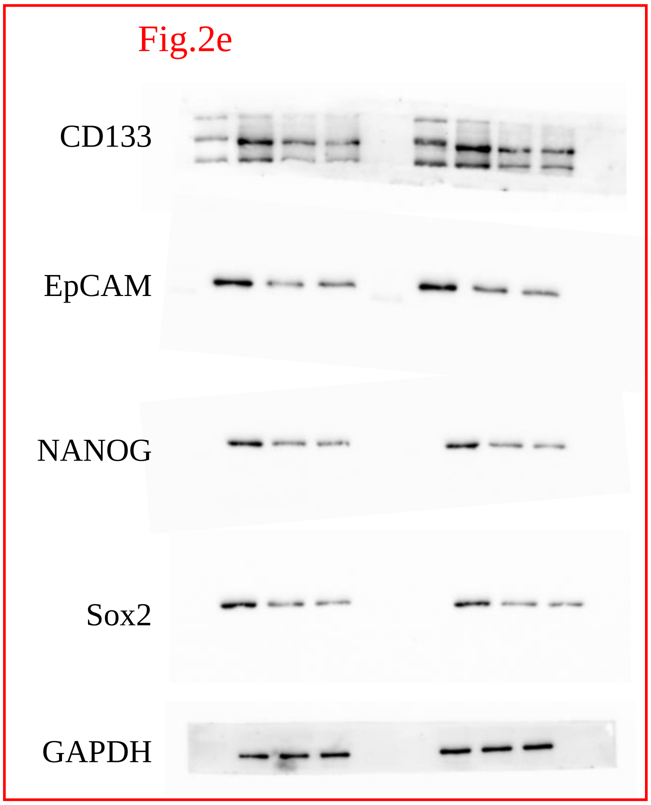

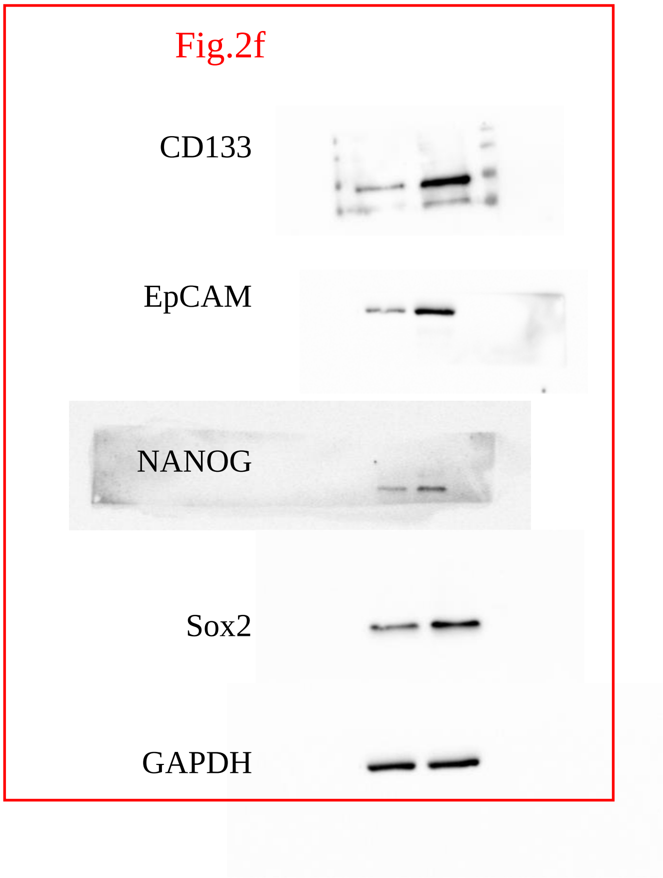

Unprocessed Wb image for Figure 5

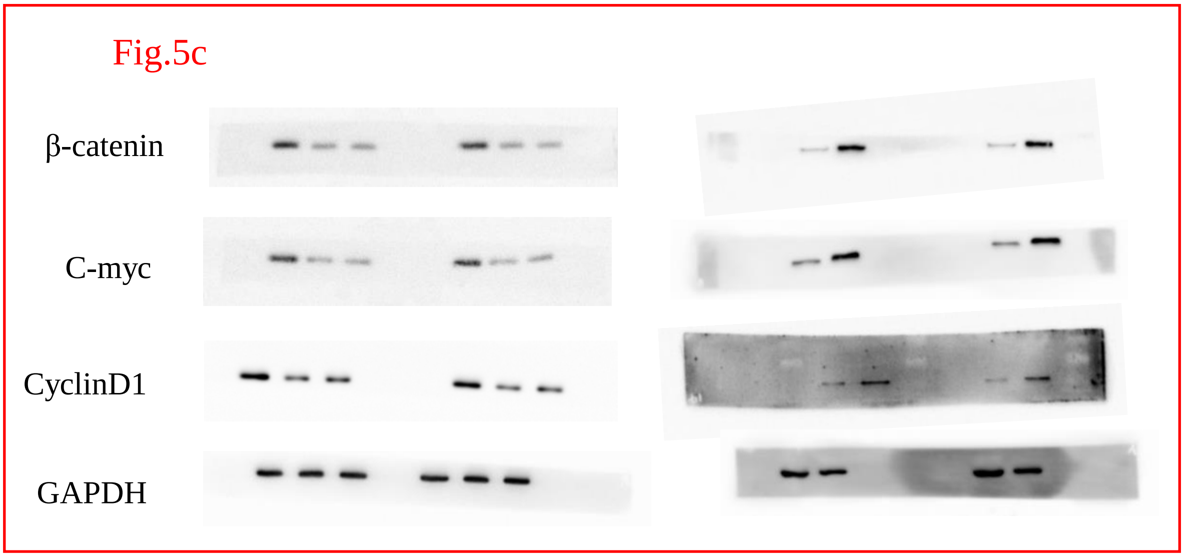

Unprocessed Wb image for Figure 6

Fig.6a

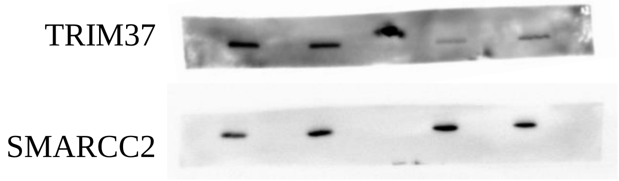

Fig.6d

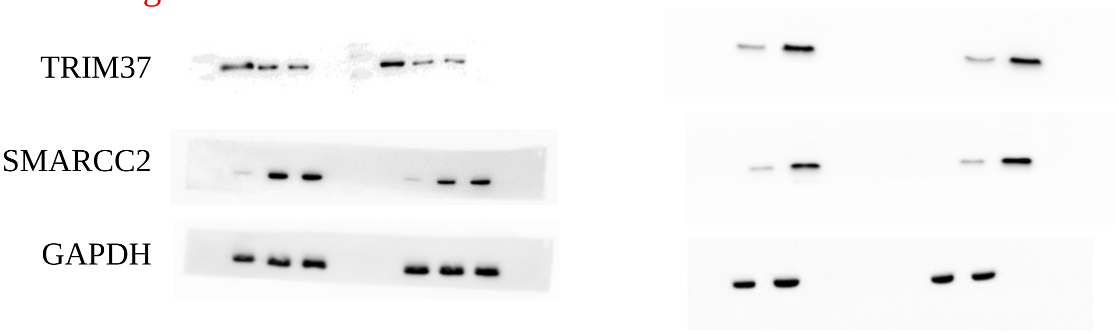

Fig.6f

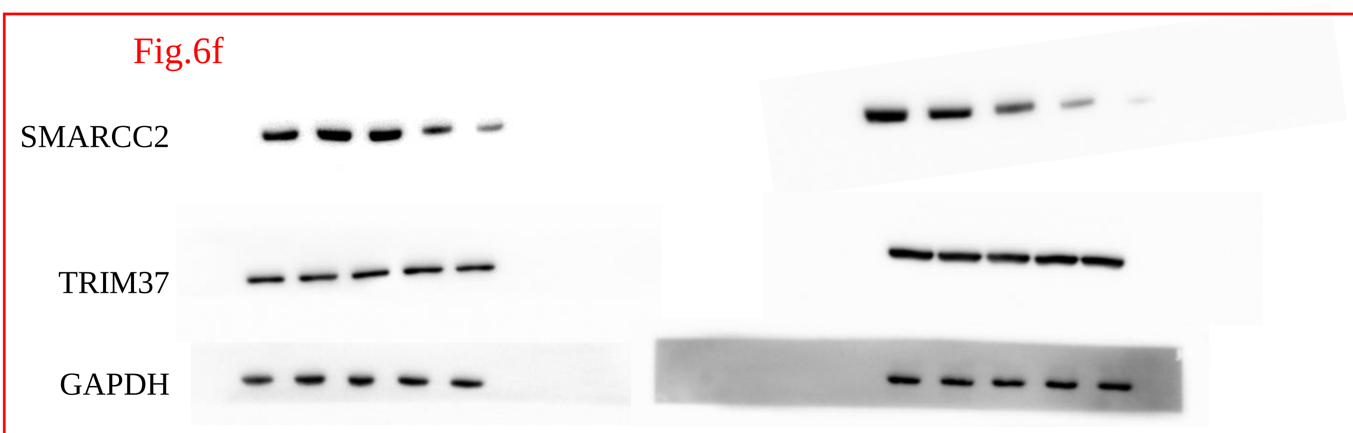

Fig.6g

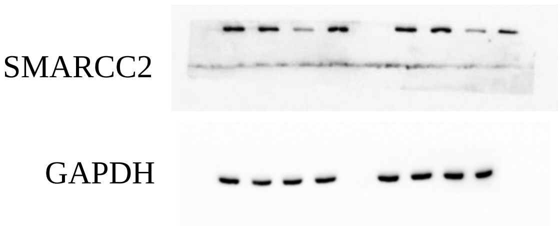

Fig.6h

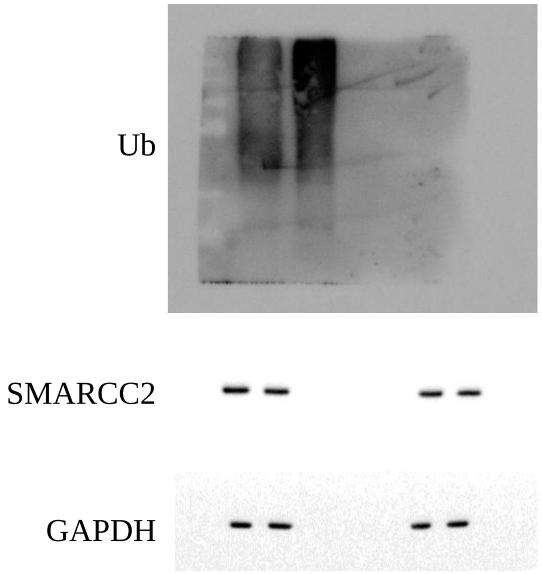

Fig.6i

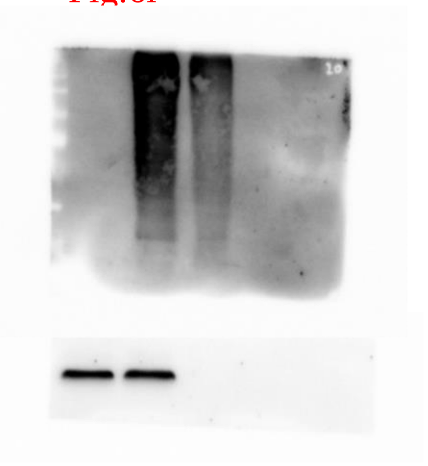

Unprocessed Wb image for Figure 7

Fig.7d

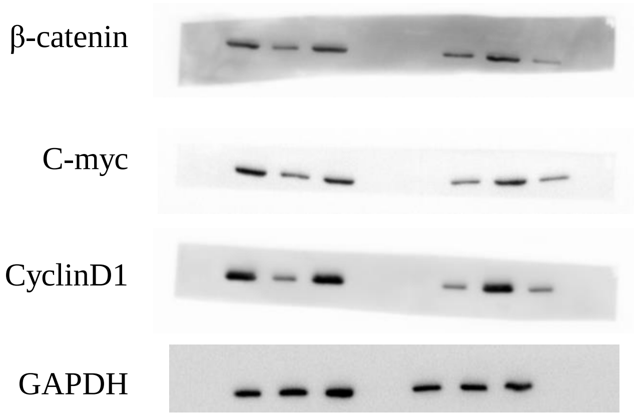

Unprocessed Wb image for Figure 8

Fig.8f

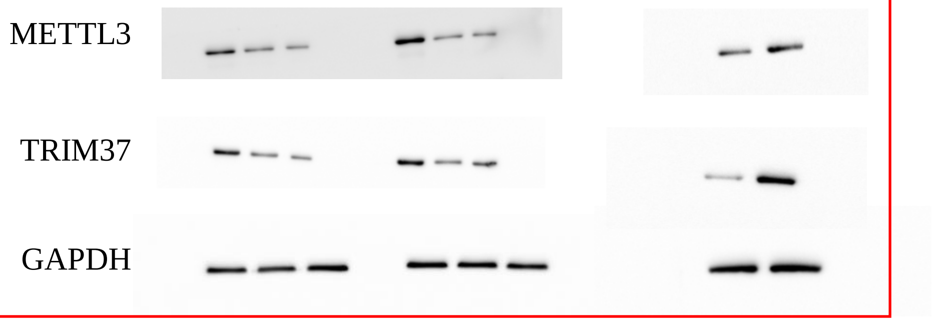

Supplement: Supplementary file 3 — Supplemantary material of WB [file 41420_2024_2187_MOESM3_ESM.pdf]
